# The efferent pathway hypothesis—A mini-review on conditioned immune enhancement

**DOI:** 10.3389/fnhum.2026.1845376

**Published:** 2026-06-25

**Authors:** Markus Rueckels, Srinivas Reddy Boreddy, Marcus Picard-Maureau

**Affiliations:** Lisa Kolk Foundation, Leverkusen, Germany

**Keywords:** anticipatory immunity, CAIP, Chrna3, conditioned immune enhancement, efferent pathway, osteopontin, Otx2, VMAT2

## Abstract

Conditioned immune responses demonstrate that learned sensory cues can modulate peripheral immunity without re-exposure to the original immunological trigger. In 2026, this research area reaches the centennial of early Pavlovian immune-reflex experiments that already reported conditioned leukocyte shifts and enhancement-like resistance to infection. Although modern psychoneuroimmunology has been shaped largely by conditioned immunosuppression, conditioned immune enhancement remains comparatively less explored despite its relevance to anticipatory host defense, immune surveillance, tumor biology, and neuroimmune regulation. This mini review focuses on the efferent, or recall, pathways of conditioned immune enhancement and separates them from afferent acquisition signals and central cue–immune-state representations. Classical odor-conditioning paradigms using camphor and the viral mimic polyinosinic:polycytidylic acid identified interferon-*β* as an acquisition-related signal, whereas recall studies implicated β-endorphin, *μ*-opioid receptor, glutamatergic/NMDA, monoaminergic, catecholaminergic, cholinergic, serotonergic, ACTH-related, interferon-*α*-related, and endocrine mechanisms. Conditioned enhancement has been shown for natural killer cell activity, cytotoxic T-lymphocyte responses, neutrophil activity, antibody responses, and tumor-model readouts. We further integrate recent circuit-level studies of insular immune-state retrieval, brain-to-spleen humoral control, vagal cytokine coding, and descending sympathetic inflammatory pathways, together with transcriptomic data from a gene-agnostic multi-tissue pilot study assessing post-recall gene-expression dynamics. Together, these findings argue against a simple hypothalamic–pituitary–adrenal axis model and instead support a temporally organized, multi-channel efferent architecture. Dissecting and understanding this emerging architecture may provide the mechanistic basis for translating the efferent arm of conditioned immune enhancement into future therapeutic concepts with clinical impact.

## Conceptual framework

Conditioned immune modulation can be defined as learned regulation of immune function by a previously neutral cue (Ader and Cohen, 1975; Hadamitzky et al., 2020; Schedlowski and Pacheco-López, 2010). In a classical conditioning paradigm, a conditioned stimulus, such as an odor, taste, or environmental context, is paired with an immune-active unconditioned stimulus, such as poly(I:C), cyclophosphamide, cyclosporine A, antigen, alloantigen, or another immunologically active compound (Ader and Cohen, 1975; Exton et al., 1998; Goebel et al., 2002; Hadamitzky et al., 2020; Hiramoto et al., 1993; Solvason et al., 1988). After acquisition, later re-exposure to the conditioned cue alone may alter peripheral immune function (Ader and Cohen, 1975; Goebel et al., 2002; Hadamitzky et al., 2020; Solvason et al., 1988; Solvason et al., 1989). The cue therefore does not directly recreate the original immune stimulus. Rather, it predicts it and recruits neural, endocrine, autonomic, and tissue-specific programs associated with the learned immune state (Hadamitzky et al., 2020; Hsueh et al., 1994; Koren et al., 2021; Schedlowski and Pacheco-López, 2010).

For conditioned immune enhancement, this predictive logic is central. While conditioned immunosuppression has clearer translational precedents, conditioned immune enhancement speaks more directly to anticipatory host defense (Ghanta et al., 1987a,b; Ghanta et al., 1988; Ghanta et al., 1990; Hiramoto et al., 1987; Solvason et al., 1988). If an organism learns that a sensory cue predicts infection, tissue damage, tumor-associated immune challenge, antigenic stimulation, or immune activation, cue re-exposure may prepare peripheral immune tissues before the full biological challenge develops (Metalnikov and Chorine, 1926; Solvason et al., 1988; Trabanelli et al., 2025). This does not imply that the brain simply “turns on” immunity in a nonspecific manner. The available evidence instead supports a temporally organized sequence involving afferent sensing, central representation, and efferent execution (Hadamitzky et al., 2020; Hsueh et al., 1994; Huerta et al., 2025; Jin et al., 2024; Kayyal et al., 2025; Koren et al., 2021; Zhang et al., 2020).

For mechanistic clarity, three levels should be separated. First, afferent acquisition pathways carry information about the immune-active unconditioned stimulus to the nervous system (Huerta et al., 2025; Jin et al., 2024; Solvason et al., 1988). Such signals may be humoral, neural, endocrine, metabolic, inflammatory, or a combination of these (Hadamitzky et al., 2020; Huerta et al., 2025; Jin et al., 2024). Second, central representation links the conditioned cue to the specific internal immune state. This is the memory component of immune conditioning (Kayyal et al., 2025; Koren et al., 2021). Third, efferent recall pathways implement the learned response in peripheral immune tissues when the organism is re-exposed to the conditioned stimulus (Hsueh et al., 1994; Solvason et al., 1989; Zhang et al., 2020).

This distinction matters because it prevents different mechanistic questions from being collapsed into one another. Afferent acquisition asks how the nervous system detects an immune-active state (Huerta et al., 2025; Jin et al., 2024; Solvason et al., 1988). Central representation asks where and how the cue-immune-state association is stored (Kayyal et al., 2025; Koren et al., 2021). Efferent recall asks how the learned immune state is re-expressed in peripheral tissues (Hsueh et al., 1994; Solvason et al., 1989; Zhang et al., 2020). The present review focuses on the third level, while integrating afferent and central mechanisms where they are necessary for interpreting the efferent pathway.

The central thesis is that conditioned immune enhancement is not adequately explained by a single hypothalamic–pituitary–adrenal axis reflex. The HPA axis is relevant, particularly because ACTH, CRH-related signaling, and splenic IFN-*α* have been implicated in the efferent pathway of conditioned NK enhancement (Hsueh et al., 1994). However, the broader view supports a multi-route efferent architecture involving endocrine, sympathetic, cholinergic, opioid, glutamatergic, catecholaminergic, serotonergic, vagal-splenic, and tissue-intrinsic decoding components (Borovikova et al., 2000; Hashimoto et al., 2026; Hsueh et al., 1994; Hsueh et al., 1995; Hsueh et al., 1996; Hsueh et al., 1999; Hsueh et al., 2002; Huerta et al., 2025; Jin et al., 2024; Kuo et al., 2001; Rosas-Ballina et al., 2011; Zhang et al., 2020).

## Historical development from immune reflexes to conditioned enhancement

The historical discovery begins around 1926, when Metalnikov and Chorine reported conditioned immune phenomena in guinea pigs and rabbits (Metalnikov and Chorine, 1926). In one experimental series, intraperitoneal immune stimulation was paired with a sensory or somatic conditioned stimulus such as scratching or local heat. After repeated pairings and a rest interval, presentation of the conditioned stimulus alone altered the peritoneal leukocyte profile (Metalnikov and Chorine, 1926). In another series, conditioned animals re-exposed to the cue before bacterial challenge showed enhancement-like resistance (Metalnikov and Chorine, 1926). Although these experiments predated modern immunological and neurobiological methodology, they already contained a principle that remains central today: a learned cue could modulate host defense capacity in both directions (Metalnikov and Chorine, 1926; Schedlowski and Pacheco-López, 2010).

The phenomenon was rediscovered by Ader and Cohen in the 1970s, who demonstrated behaviorally conditioned immunosuppression of a specific antibody response to a defined antigen by pairing a taste cue with cyclophosphamide (Ader and Cohen, 1975). Their work had major conceptual impact because it showed that learned cues can alter peripheral immune function without re-administration of the original immunopharmacological agent (Ader and Cohen, 1975; Hadamitzky et al., 2020; Schedlowski and Pacheco-López, 2010). While initially facing strong skepticism from parts of the established scientific community, it also shaped the subsequent direction of the field. Conditioned immunosuppression became the more investigated branch, partly because it could be reliably replicated in animal models and later extended to human placebo-conditioning paradigms (Exton et al., 1998; Goebel et al., 2002; Hadamitzky et al., 2020; Schedlowski and Pacheco-López, 2010). A human cyclosporine A conditioning study then showed that pairing an immunosuppressive drug with a flavored drink can condition suppression of IL-2 and IFN-*γ* mRNA expression, cytokine production, and lymphocyte proliferation after cue re-exposure in a clinical context (Goebel et al., 2002).

While this historical bias toward immunosuppression is understandable, it should not obscure the parallel development of conditioned immune enhancement. From the late 1980s onward, Nancy S. and Raymond N. Hiramoto, together with Vithal K. Ghanta, H. Brent Solvason, Chi-Mei Hsueh, and colleagues, developed a series of enhancement paradigms in which learned cues increased NK-cell activity, cytotoxic T-lymphocyte activity, neutrophil activity, and tumor-relevant immune effects (Chao et al., 2005; Ghanta et al., 1987a,b; Ghanta et al., 1988; Ghanta et al., 1990; Hiramoto et al., 1993; Hiramoto et al., 1987; Hsueh et al., 1994; Solvason et al., 1988). These studies not only showed that conditioning can be translated into measurable immune enhancement but also paved the way for separating the acquisition process from the central cue-immune-state association and the efferent route that implements recall (Ghanta et al., 1987b; Hiramoto et al., 1987; Hsueh et al., 1994; Solvason et al., 1988; Solvason et al., 1989).

One of the earliest enhancement paradigms paired exposure to camphor odor as conditioned stimulus with poly(I:C) as immune-active unconditioned stimulus (Ghanta et al., 1987b; Hiramoto et al., 1987; Solvason et al., 1988). Poly(I:C) is a synthetic double-stranded RNA analog that mimics viral infection and induces type I interferon responses (Solvason et al., 1988). After acquisition, animals were later re-exposed to camphor odor alone, and NK-cell activity was used as the recall readout (Ghanta et al., 1987b; Solvason et al., 1988). Solvason, Ghanta, and Hiramoto showed that conditioned NK enhancement was not explained by nonspecific nociceptive properties of the cue (Solvason et al., 1988). They also showed that IFN-*β*, but not IFN-*α*, could substitute for poly(I:C) as the unconditioned stimulus (Solvason et al., 1988). This was a key acquisition-stage finding: IFN-β functions as a learning signal, whereas recall could not be reduced to a generic peripheral interferon surge (Ghanta et al., 1987b; Solvason et al., 1988).

## Classical enhancement paradigms and staged mechanism

The early conditioned immune enhancement literature developed stepwise. First, it showed that conditioned immune responses are directionally programmable (Ghanta et al., 1987b; Hiramoto et al., 1987). Depending on the immune-active unconditioned stimulus, the same general conditioning logic could enhance or suppress immune activity (Ader and Cohen, 1975; Ghanta et al., 1987b; Hiramoto et al., 1987; Solvason et al., 1988). Poly(I:C) supported conditioned enhancement, whereas cyclophosphamide supported suppression (Ader and Cohen, 1975; Solvason et al., 1988). This placed immune enhancement within the same Pavlovian framework as conditioned immunosuppression while showing that the immune outcome depends on the internal state paired with the cue (Hadamitzky et al., 2020; Hiramoto et al., 1987; Schedlowski and Pacheco-López, 2010).

Second, the camphor/poly(I:C) model was used to identify the acquisition, or so-called afferent, pathway. IFN-*β* could replace poly(I:C) as one of the unconditioned stimuli, whereas IFN-*α* could not (Solvason et al., 1988). This distinction is important because IFN-β appears to participate in acquisition, while IFN-α later appears in the recall literature as a splenic mediator linked to efferent endocrine output (Hsueh et al., 1994; Solvason et al., 1988). The key point is therefore not that “interferon” generically explains conditioned immune enhancement. Rather, different interferons may be participating with different roles at different stages of the conditioning sequence (Ghanta et al., 1987b; Hsueh et al., 1994; Solvason et al., 1988).

Third, work shifted from acquisition to efferent expression. Naltrexone blocked expression of conditioned NK enhancement when given before cue re-exposure, suggesting that recall required an opioid-sensitive mechanism (Solvason et al., 1989). Related CTL work supported a central component because studies with naltrexone and quaternary naltrexone pointed to a CNS-dependent recall pathway involving opioid receptors (Hiramoto et al., 1993). Subsequent studies refined this model by showing *β*-endorphin dependence and a requirement for *μ*-opioid receptor activation (Hsueh et al., 1995; Hsueh et al., 1996).

Fourth, endocrine outputs were tested directly. Again, in the camphor/poly(I:C) paradigm, plasma *β*-endorphin and ACTH were quantified, while splenic interferon message was assessed (Hsueh et al., 1994). ACTH and splenic IFN-*α* gene expression were higher in conditioned animals than in controls (Hsueh et al., 1994). Dexamethasone blocked expression of conditioned NK enhancement without preventing acquisition (Hsueh et al., 1994). Together, these findings supported an HPA-linked efferent arm in which pituitary ACTH may contribute to splenic IFN-*α* upregulation and NK activation (Hsueh et al., 1994).

Fifth, recall-stage neurotransmission was dissected further. Reserpine and other catecholamine-related interventions disrupted conditioned NK enhancement, implicating monoamine and catecholamine signaling (Hiramoto et al., 1990; Hsueh et al., 1999). Later work showed that glutamate and NMDA receptors, but not GABA or AMPA/kainate receptors, were required for recall (Kuo et al., 2001). A parallel extension beyond NK biology came from conditioned neutrophil enhancement, where catecholamine depletion with reserpine or 6-hydroxydopamine blocked recall, whereas dexamethasone did not produce a comparable blockade (Chao et al., 2005). Thus, different immune effector systems may rely on different combinations of endocrine, opioid, glutamatergic, monoaminergic, and catecholaminergic efferent signals (Chao et al., 2005; Hiramoto et al., 1990; Hsueh et al., 1994; Hsueh et al., 1999; Hsueh et al., 2002; Kuo et al., 2001).

Taken together, the older enhancement studies identified several recall-stage messengers and checkpoints: endogenous opioids, *β*-endorphin-related peptides, *μ*-opioid receptors, glutamate/NMDA signaling, monoamine/catecholamine handling, cholinergic and serotonergic signaling, ACTH, IFN-*α*, and endocrine routing (Hiramoto et al., 1990; Hsueh et al., 1994; Hsueh et al., 1995; Hsueh et al., 1996; Hsueh et al., 1999; Hsueh et al., 2002; Kuo et al., 2001; Solvason et al., 1989). Importantly, these were not transcriptomic associations. They were functional, pharmacological, endocrine, and immunological findings. Their strength is that they tested necessity or association during recall rather than merely listing candidate molecules (Hsueh et al., 1994; Hsueh et al., 1995; Hsueh et al., 1999; Kuo et al., 2001; Solvason et al., 1989).

## Extension beyond NK cells

While conditioned immune enhancement in its early beginnings was mostly focused on NK-cell biology, Hiramoto and colleagues in parallel started to use conditioned allogeneic cytotoxic T-lymphocyte responses by pairing camphor odor with C57BL/6 spleen-cell immunization in BALB/c mice (Hiramoto et al., 1993). Recall of this acquired cytotoxic response was blocked by centrally active naltrexone but not by quaternary naltrexone, supporting the involvement of central opioid receptors in expression of this T-cell-mediated response (Hiramoto et al., 1993).

Conditioned enhancement was also extended to neutrophil activity (Chao et al., 2005). In this paradigm, neutrophil activity increased after conditioned training, and catecholamine depletion blocked the conditioned innate immune response at the recall stage (Chao et al., 2005). This finding is important because it suggests that catecholaminergic efference may be particularly relevant for some innate effector systems, and that HPA-axis blockade alone may not explain the full recall architecture (Chao et al., 2005; Hsueh et al., 1994; Hsueh et al., 1999).

Conditioned enhancement of antibody responses provides a further extension. Additional one-trial conditioning work by Madden and colleagues showed conditioned enhancement of the antibody response to hen egg lysozyme in rats, further supporting antibody production as an immune-effector domain in which conditioned enhancement can be observed (Madden et al., 2000; Madden et al., 2001). Antigen-paired taste or odor cues can enhance later antibody production, and lesion studies indicate that insular cortex and amygdala lesions disrupt conditioned antibody enhancement, whereas hippocampal lesions do not show the same effect (Alvarez-Borda et al., 1995; Alvarez-Borda et al., 1999). These data pointed toward central acquisition and representation structures long before modern engram and circuit tools became available (Alvarez-Borda et al., 1995; Alvarez-Borda et al., 1999; Kayyal et al., 2025; Koren et al., 2021).

The important conclusion is that conditioned immune enhancement is a specific, immune-system-level phenomenon, not a single NK-cell assay artifact. Different effector compartments may share the general architecture of cue-based recall while relying on distinct peripheral implementation routes (Alvarez-Borda et al., 1995; Alvarez-Borda et al., 1999; Chao et al., 2005; Ghanta et al., 1987b; Ghanta et al., 1990; Hiramoto et al., 1993; Hiramoto et al., 1987; Hsueh et al., 1994; Solvason et al., 1988; Solvason et al., 1989).

## Tumor-model evidence and translational relevance

The tumor-model work gives the early conditioned immune enhancement literature additional translational relevance. In MOPC 104E myeloma-bearing mice, camphor/poly(I:C) conditioning was associated with increased median survival after cue re-exposure (Ghanta et al., 1987a; Ghanta et al., 1988). In related work, plastic-adherent spleen cells, later interpreted as macrophage-enriched cells, suppressed tumor IgM production by MOPC cells and reduced colony formation *in vitro* (Ghanta et al., 1988). These findings suggested that conditioned enhancement may involve macrophage-like effector mechanisms even in tumor-relevant paradigms (Ghanta et al., 1987a; Ghanta et al., 1988).

In the YC8 T-cell lymphoma model, camphor odor was paired with allogeneic DBA/2 spleen-cell immunization in BALB/c mice bearing syngeneic YC8 lymphoma (Ghanta et al., 1990). After repeated CS/US pairings, conditioned animals were re-exposed to camphor odor alone (Ghanta et al., 1990). The conditioned groups showed delayed tumor growth and conditioned immunotherapeutic activity (Ghanta et al., 1990). While these *in vivo* studies remain preclinical, they already demonstrated in the 1990s that conditioned immune enhancement can be used as a precursor for translational immunotherapy research (Ghanta et al., 1987a; Ghanta et al., 1988; Ghanta et al., 1990).

The main point here is not that these early studies establish a ready clinical intervention. They do not. Rather, they show that conditioned immune enhancement can be used to identify molecular pathways and messengers that affect disease-relevant immune endpoints *in vivo* (Ghanta et al., 1987a; Ghanta et al., 1988; Ghanta et al., 1990). This makes the efferent pathway question more than a mechanistic curiosity. If learned cues can alter anti-tumor immune activity in animal models, then identifying the neural, endocrine, autonomic, and tissue-intrinsic routes of recall becomes an interesting future opportunity to identify drug candidates that might be able to mimic the immune-enhancement effect in a specific, safe, and clinically meaningful manner (Ben-Shaanan et al., 2016; Ben-Shaanan et al., 2018; Ghanta et al., 1987a; Ghanta et al., 1988; Ghanta et al., 1990; Hsueh et al., 1994).

In this context, perioperative oncology work by Ben-Eliyahu and colleagues may be interpreted more narrowly. Their core research does not focus on conditioned immune enhancement. However, it identifies a clinically relevant efferent context: surgery and stress during cancer-related surgery can increase catecholamines and prostaglandins, suppress anti-metastatic cell-mediated immunity, and influence metastasis-relevant biology, which is a major determinant of long-term outcome after tumor resection surgery (Ben-Eliyahu, 2003; Horowitz et al., 2015). This supports the broader point that neuroendocrine efferent states may matter more in clinical oncology than currently considered (Ben-Eliyahu, 2003; Ben-Shaanan et al., 2018; Horowitz et al., 2015).

## Central representation and anticipatory recall

Following acquisition, conditioned immune enhancement requires the cue-immune association to be stored in the nervous system (Hadamitzky et al., 2020; Hiramoto et al., 1993; Kayyal et al., 2025; Koren et al., 2021; Solvason et al., 1989). Odor specificity implies sensory cue representation through olfactory pathways (Ghanta et al., 1987b; Solvason et al., 1988). The persistence of conditioned recall implies memory storage, a hypothesis initially supported by the fact that centrally active opioid blockade interferes with recall and later reinforced by circuit-level work on immune retrieval (Hiramoto et al., 1993; Kayyal et al., 2025; Koren et al., 2021; Solvason et al., 1989).

Recent neuroimmunology now gives this older concept a more explicit anatomical and circuit-level model. Koren and colleagues showed in 2021 that neuronal ensembles in the mouse insular cortex activated during distinct inflammatory conditions can both retrieve or suppress associated peripheral immune responses (Koren et al., 2021). This provides direct support for the idea that immune states can be centrally represented and later retrieved (Koren et al., 2021).

Kayyal and colleagues later linked retrieval of conditioned immune responses to a bidirectional anterior–posterior insula circuit (Kayyal et al., 2025). In that study, anterior-to-posterior insula projections supported behavioral retrieval, while bidirectional anterior–posterior interactions contributed to the anticipatory immunological component (Kayyal et al., 2025). This finding is particularly relevant because it connects conditioned immune retrieval to a defined central circuit rather than to a generic “brain effect” (Kayyal et al., 2025).

This framework clarifies the meaning of anticipatory immunity. A conditioned cue does not merely refer to a past immune event. Once learned, it becomes specifically predictive. Cue re-exposure can therefore be interpreted as a central prediction that a biologically relevant immune state may recur (Kayyal et al., 2025; Koren et al., 2021; Trabanelli et al., 2025). Upon recall, the organism may recruit selected efferent pathways that bias peripheral tissues toward readiness. This is conceptually different from a full inflammatory replay. A plausible model is selective anticipatory preparation: central recall activates peripheral immune components that may improve readiness while limiting the metabolic and tissue-damaging costs of full infection-like inflammation (Kayyal et al., 2025; Koren et al., 2021; Trabanelli et al., 2025).

Human work on anticipated infection cues is adjacent to this idea. Virtual infection-cue studies are not classical CS/US conditioning paradigms, but they show that perceived infectious threat can engage salience-related brain systems and alter immune readouts even in the absence of real infection (Trabanelli et al., 2025). Such findings support the broader principle of anticipatory neuroimmune regulation, while conditioned immune suppression or enhancement remain the more experimentally precise Pavlovian models (Hadamitzky et al., 2020; Kayyal et al., 2025; Koren et al., 2021; Schedlowski and Pacheco-López, 2010; Trabanelli et al., 2025).

## Modern efferent pathway context

Modern neuroimmunology supports a multi-route efferent model rather than a single endocrine reflex. The HPA axis remains relevant, particularly because ACTH and splenic IFN-*α* are implicated in classical conditioned enhancement (Hsueh et al., 1994). However, newer work shows that central immune control can be routed through distinct and complementary neural and neuroendocrine pathways (Ben-Shaanan et al., 2016; Ben-Shaanan et al., 2018; Borovikova et al., 2000; Hashimoto et al., 2026; Huerta et al., 2025; Jin et al., 2024; Kayyal et al., 2025; Koren et al., 2021; Rosas-Ballina et al., 2011; Zhang et al., 2020).

## Reward-system modulation of immunity

Groundbreaking work by Asya Rolls and colleagues showed that activation of central reward circuitry can enhance innate and adaptive immune functions (Ben-Shaanan et al., 2016). Chemogenetic activation of the ventral tegmental area strengthened antibacterial host defense and heightened T-cell responses, with effects at least partly mediated by the sympathetic nervous system (Ben-Shaanan et al., 2016). Subsequent studies showed that activation of the brain’s reward system attenuated tumor growth in Lewis lung carcinoma and B16 melanoma models through neuroimmune mechanisms involving sympathetic regulation and myeloid-derived suppressor cells (Ben-Shaanan et al., 2018). While these studies are not classical immune-conditioning experiments, they demonstrate that defined central neural states can modulate peripheral immune and tumor-relevant outcomes in a structured manner (Ben-Shaanan et al., 2016; Ben-Shaanan et al., 2018).

## Brain-to-spleen control of humoral immunity

Taking it further, Zhang and colleagues provided a causal circuit-level example of brain-to-spleen immune control (Zhang et al., 2020). Their work identified a brain-spleen neural connection that autonomically enhances humoral immune responses and suggested that immune stimulation can be influenced by behavior (Zhang et al., 2020). The described pathways involve central CRH-related circuitry, splenic nerve activity, acetylcholine-producing T-cell relay, and nicotinic signaling on B cells, further demonstrating a brain-to-spleen route that is not reducible to a purely hormonal HPA cascade (Rosas-Ballina et al., 2011; Zhang et al., 2020).

## Cholinergic and vagal-splenic relay logic

Another initially seemingly unrelated path, the inflammatory reflex and cholinergic anti-inflammatory pathway (CAIP) literature, shows that vagus nerve stimulation can attenuate systemic inflammatory responses to endotoxin (Borovikova et al., 2000). Later work demonstrated that acetylcholine-synthesizing T cells relay neural signals in a vagus nerve circuit and are required for inhibition of cytokine production by vagus nerve stimulation (Rosas-Ballina et al., 2011). These findings establish that immune cells can serve as intermediaries in neuroimmune communication rather than merely as passive targets of neural output (Borovikova et al., 2000; Rosas-Ballina et al., 2011; Zhang et al., 2020).

In the conditioned recall transcriptomic map discussed below, Chrna3, encoding the nicotinic acetylcholine receptor α3 subunit, appears as part of the splenic recall signature. While this does not prove direct vagal causality in conditioned immune enhancement, it fits with a broader cholinergic relay model and provides a plausible molecular bridge between splenic recall and nicotinic decoding (Rosas-Ballina et al., 2011; Zhang et al., 2020).

## Cytokine-specific afferent coding and descending inflammatory output

Recent body–brain studies have also begun to remap cytokine-specific afferent channels. Jin and colleagues showed that pro-inflammatory and anti-inflammatory cytokines communicate with distinct populations of vagal neurons and that this body–brain circuit modulates peripheral inflammatory balance (Jin et al., 2024). Additionally, Huerta and colleagues used *in vivo* calcium imaging to show that vagal sensory neurons within the nodose ganglia exhibit distinct real-time neuronal responses to inflammatory cytokines (Huerta et al., 2025). These studies are primarily afferent rather than efferent, but they are relevant to conditioned immune enhancement because acquisition requires immune-state information to reach the nervous system (Huerta et al., 2025; Jin et al., 2024; Solvason et al., 1988).

Hashimoto and colleagues then added a complementary efferent example by showing that CRH-expressing BNST neurons encode IL-1*β* signals and orchestrate cardiovascular and inflammatory responses through a BNST → PVN → RVLM → β-adrenergic pathway (Hashimoto et al., 2026). This was not identified by a conditioned immune-enhancement paradigm, but it demonstrates that inflammatory information can be represented centrally and routed back to the periphery through defined descending sympathetic pathways (Hashimoto et al., 2026).

## Multi-tissue transcriptomics analysis as a supporting efferent map

A 2025 transcriptomic pilot study from our laboratory extended the efferent pathway question by applying pathway-agnostic gene-expression analysis across candidate recall tissues. Rats were conditioned using camphor smell as conditioned stimulus and intraperitoneal poly(I:C) as unconditioned stimulus. After a 48-h interval, test animals were re-exposed to camphor smell with saline rather than poly(I:C); positive controls received poly(I:C) re-injection; and negative controls received smell exposure with saline. Hypothalamus, pituitary, adrenal glands, and spleen were collected at 0, 3, 6, 24, and 48 h after recall or control procedure. Tissue RNA was analyzed using rat genome arrays, selected genes were validated by qRT-PCR, and plasma proteins were analyzed by Luminex.

**Figure 1:**
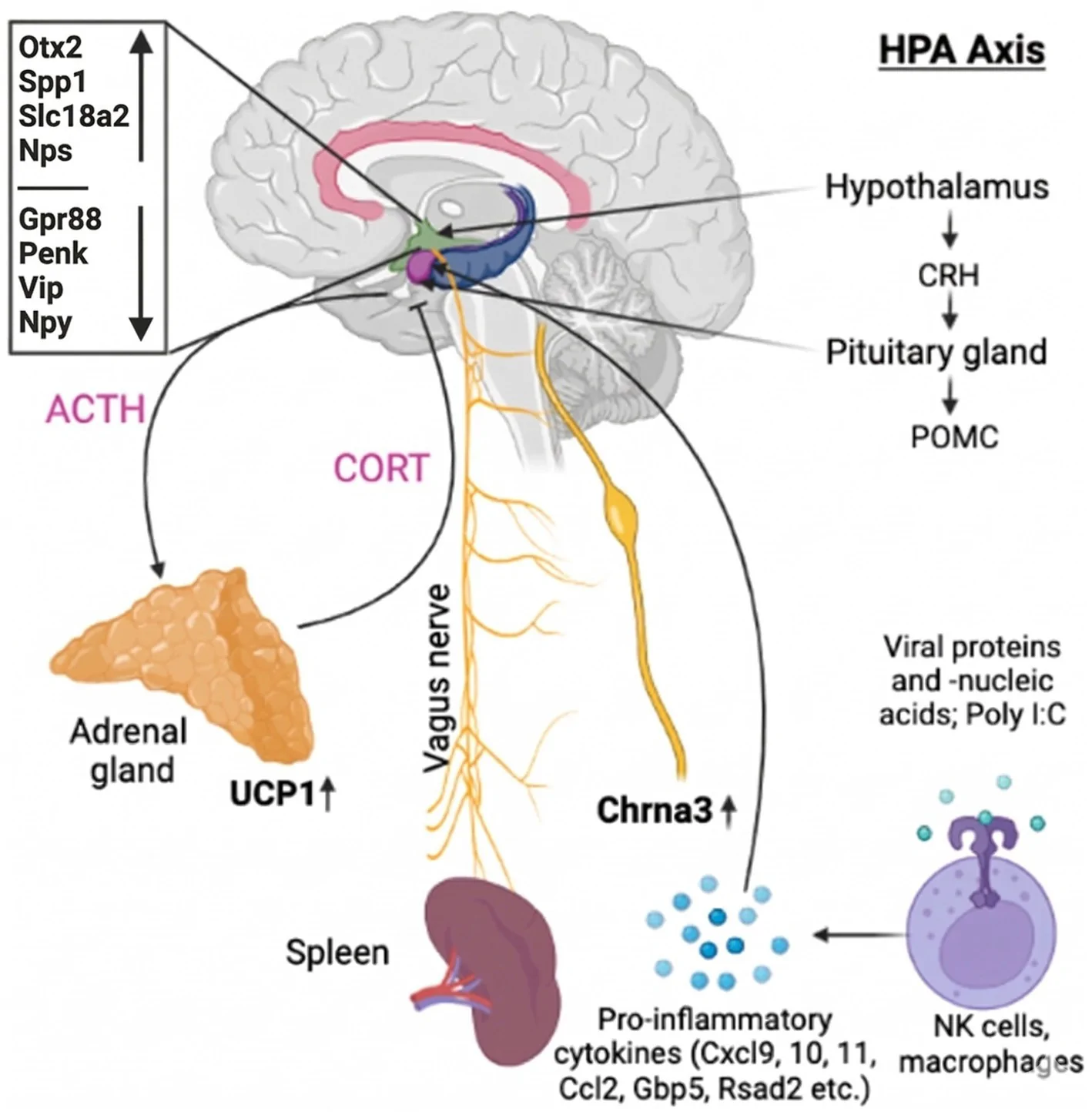
Candidate efferent pathway map for conditioned immune enhancement after camphor/poly(I:C) recall, based on a multi-tissue transcriptomic and plasma pilot study and contextualized by established neuroimmune relay pathways. The schematic summarizes potential hypothalamic, HPA-axis, adrenal, vagal-splenic, and immune-cell-associated candidate genes, mediators, and pathway components, including Otx2, Spp1/osteopontin (OPN), Slc18a2/VMAT2, Chrna3-linked splenic nicotinic signaling, and CAIP-related vagal-splenic relay logic. Adapted from Rueckels and Picard-Maureau (2025).

While these datasets should be carefully interpreted as hypothesis-generating rather than causal proof, this pathway-agnostic approach aligns directly with recent unbiased, multi-tissue omics profiling of the neuroendocrine-immune network. For example, Zeng and colleagues conducted a multi-tissue transcriptomic analysis of the hypothalamus, adrenal glands, and spleen after high-thoracic spinal cord injury, demonstrating how neuroinflammation suppresses hypothalamic Gi-mediated GPCR signaling, including Grm4, Nmu, and Chrm2, and disrupts the HPA-axis connection to drive adrenal circadian-rhythm dysfunction, including Per2, Per3, and Cry1, and splenic immune failure. This independent example supports the rationale for using pathway-agnostic cross-tissue transcriptomics to map integrated neuroendocrine-immune efferent architecture. For the present review, the most relevant findings from our pilot study concern the hypothalamic recall-candidate cluster and the splenic immune-decoding signature (Zeng et al., 2023) (Figure 1).

## Hypothalamic recall candidates

In the hypothalamus, the most relevant recall cluster centers on Otx2, Spp1, and Slc18a2 (Rueckels and Picard-Maureau, 2025). Otx2 is a homeobox transcription factor associated with developmental and transcriptional regulation (Rueckels and Picard-Maureau, 2025). Spp1 encodes osteopontin, a secreted phosphoprotein involved in immune regulation, extracellular-matrix biology, inflammation, tissue remodeling, and oncological contexts (Mahmud et al., 2020; Rueckels and Picard-Maureau, 2025). Slc18a2 encodes vesicular monoamine transporter 2, or VMAT2, which participates in vesicular storage of dopamine, norepinephrine, serotonin, and related monoamines (Rueckels and Picard-Maureau, 2025). Together, this triad connects well to earlier findings of conditioned recall involving transcriptional regulation, immune-neural matrix signaling, and monoaminergic neurotransmitter signaling (Hiramoto et al., 1990; Hsueh et al., 1999; Rueckels and Picard-Maureau, 2025).

Among these genes, Spp1/osteopontin deserves contextualization because it provides a plausible link between immune regulation and neuroinflammatory restraint (Mahmud et al., 2020; Rueckels and Picard-Maureau, 2025). In a chronic viral-neuroinflammation model, osteopontin/secreted phosphoprotein-1 was described as a molecular brake on neuroinflammatory responses (Mahmud et al., 2020). While this does not prove a causal role for osteopontin in conditioned immune enhancement, it makes the hypothalamic Spp1 signal plausible as one candidate modulator linking central immune integration, neuroinflammatory regulation, and downstream endocrine or autonomic routing (Mahmud et al., 2020; Rueckels and Picard-Maureau, 2025).

The same pilot dataset reported Wnt/*β*-catenin-related candidates including Fzd6 and Zic1, broader Sox-family involvement, and recall-associated regulation of dopaminergic, serotonergic, and neuropeptide-related genes such as Drd1, Drd2, Htr1f, Htr2a, Vip, Pmch, Npy, and Nps (Rueckels and Picard-Maureau, 2025). Opioid-related and neuropeptide-processing candidates such as Gpr88, Gpr52, Penk, Pnoc, Pcsk1, and Pcsk2 connect the transcriptomic map to the older pharmacological evidence that naltrexone, *β*-endorphin, *μ*-opioid receptors, and neuropeptide processing participate in recall (Hsueh et al., 1994; Hsueh et al., 1995; Hsueh et al., 1996; Rueckels and Picard-Maureau, 2025; Solvason et al., 1989). These data are therefore in line with several circuits and messenger systems identified decades earlier (Hiramoto et al., 1990; Hsueh et al., 1994; Hsueh et al., 1995; Hsueh et al., 1996; Hsueh et al., 1999; Hsueh et al., 2002; Kuo et al., 2001; Solvason et al., 1989).

## Splenic recall candidates

While transcriptomic analysis also identified candidate genes in pituitary and adrenal glands, the splenic recall signature complements the hypothalamic pattern (Rueckels and Picard-Maureau, 2025). Upregulated splenic markers included immune-modulating transcripts such as MOR5S4 (“Hiramoto factor”), Ly6al, Isg15, Ifitm3, Ifit3, Chrna3, and Fut7 (Rueckels and Picard-Maureau, 2025). qRT-PCR validation further included immune and inflammatory candidates such as Ccl12, Ccl2, Ccl5, Cd274, Cx3cl1, Cxcl9, Cxcl10, Cxcl11, Fosl2, Gbp5, Il10, Il1a, Il1b, Il21, Irf1, Irf7, Junb, Ly49si1, Mmp12, Mx1, Mx2, Rsad2, RT1-A1, and Tnf (Rueckels and Picard-Maureau, 2025). This places the spleen within an interferon-, chemokine-, and cytotoxicity-related recall program (Hu, 2020; Rueckels and Picard-Maureau, 2025).

The splenic neurotransmission-related signature also carries interesting findings. The study reported upregulation of Pcsk1, Pcsk2, Sigmar1, Chrna3, Grid2, Grin1a, Slc1a4, and Gad2, and downregulation of Oprd1, Chrm2, Grik1, Grm4, Gabbr1, Gabrp, and Htr1d (Rueckels and Picard-Maureau, 2025). This supports a model in which the spleen is not merely a passive endocrine target. Rather, it may act as a decoding organ that integrates endocrine, cholinergic, glutamatergic, opioid-related, cytokine-related, and local immune signals in line with findings from research published more than three decades ago (Hsueh et al., 1994; Rosas-Ballina et al., 2011; Rueckels and Picard-Maureau, 2025; Zhang et al., 2020).

In this context, Chrna3 is particularly noteworthy. While it should not be overinterpreted as proof of a direct vagal efferent mechanism, its transient upregulation in the spleen after recall would be consistent with cholinergic relay logic and with the broader literature on neural regulation of splenic immunity (Rosas-Ballina et al., 2011; Rueckels and Picard-Maureau, 2025; Zhang et al., 2020).

## Plasma mediators and temporal propagation

Plasma measurements provided an additional humoral layer. The Luminex panel included ACTH, BDNF, as well as multiple cytokines and chemokines (Rueckels and Picard-Maureau, 2025). ACTH was found elevated during the first 6 hours after recall in both test and positive-control animals, providing continuity with the older ACTH-focused efferent-signal work (Hsueh et al., 1994; Rueckels and Picard-Maureau, 2025). IFN-*γ* increased immediately after recall in the test condition and from 0 to 3 h in positive controls (Rueckels and Picard-Maureau, 2025). Pronounced chemokine changes were also reported for IP-10/CXCL10, MCP-1/CCL2, MIP-1*α*/CCL3, RANTES/CCL5, and TNF-α, with a strong peak at approximately 6 h after recall (Rueckels and Picard-Maureau, 2025).

While seemingly complex, this temporal structure may be of interest, too. Several test-group recall changes appeared delayed relative to direct poly(I:C) re-stimulation by approximately 3 hours (Rueckels and Picard-Maureau, 2025). This supports the idea that cue-only recall is not an instantaneous peripheral mimicry of poly(I:C) but may first activate central programs and only later then propagate into endocrine, autonomic, humoral, and splenic transcriptional decoding (Rueckels and Picard-Maureau, 2025). This sequence would be precisely in line with the temporal order predicted by an efferent pathway model (Hsueh et al., 1994; Rueckels and Picard-Maureau, 2025).

Limitations are equally important. The pilot study had limited sample size, was constrained by tissue-level rather than cell-type-specific readouts and did not establish anatomical necessity or causal direction (Rueckels and Picard-Maureau, 2025). It also cannot by itself distinguish every conditioned effect from possible residual immune activation or handling-related confounds (Rueckels and Picard-Maureau, 2025). Because of these limitations, all genes identified as regulated in this pilot study should be considered testable candidates for future studies rather than a proof of mechanism (Rueckels and Picard-Maureau, 2025).

## Immunological interpretation through type I interferon and the THα*β* framework

The immunological pattern of poly(I:C)-conditioned enhancement is most coherently interpreted through type I interferon-linked antiviral preparedness (Hu, 2020; Solvason et al., 1988). Poly(I:C) mimics viral double-stranded RNA and activates innate antiviral signaling (Solvason et al., 1988). In classical conditioning literature, IFN-β could substitute for poly(I:C) as an acquisition-stage unconditioned stimulus, whereas IFN-*α* appeared later as a recall-associated splenic mediator (Hsueh et al., 1994; Solvason et al., 1988). This implies stage-specific interferon involvement rather than a generic interferon explanation (Ghanta et al., 1987b; Hsueh et al., 1994; Solvason et al., 1988).

Interestingly, at the splenic level, the pilot gene-expression map after recall strongly overlaps with a partial antiviral-like pattern (Hu, 2020; Rueckels and Picard-Maureau, 2025). Candidate genes include Irf7, Isg15, Ifitm3, Ifit3, Gbp2, Gbp5, Igtp, Mx2, Rsad2, and the CXCR3-ligand chemokines Cxcl9, Cxcl10, and Cxcl11 (Hu, 2020; Rueckels and Picard-Maureau, 2025). These markers suggest that cue-only recall may engage selected antiviral and chemokine elements without reproducing the full inflammatory architecture of actual viral infection (Hu, 2020; Rueckels and Picard-Maureau, 2025).

As one possible explanation for the observed gene regulation, the so-called THαβ concept by Hu and colleagues may provide an immunological framework for this pattern, especially because it links antiviral-type immunity to type I interferon-related signaling, nucleic-acid-sensing TLR pathways, CXCR3-linked chemokines, NK cells, cytotoxic CD8 T cells, and selected B-cell responses (Hu, 2020). The THαβ framework should therefore be treated as a potential interpretive immunological framework in future studies for the observed recall pattern rather than as a proven mechanism of conditioned immune enhancement (Hu, 2020; Rueckels and Picard-Maureau, 2025; Solvason et al., 1988).

## Operationalized efferent pathway hypothesis

The most useful way to formulate the efferent pathway hypothesis is as an operational map for future research. Conditioned immune enhancement can be modeled as a sequence in which immune-active acquisition signals are first encoded through afferent channels, then associated with cue-specific central representations, and finally translated into peripheral immune states through multiple efferent routes (Hadamitzky et al., 2020; Hsueh et al., 1994; Huerta et al., 2025; Jin et al., 2024; Kayyal et al., 2025; Koren et al., 2021; Rueckels and Picard-Maureau, 2025; Solvason et al., 1988; Zhang et al., 2020).

In concrete terms, the candidate network includes cytokine-sensing vagal afferents in the nodose ganglia, brainstem cNST/DBH neurons that shape inflammatory balance, insula-based immune-state representation and anterior–posterior insula retrieval circuitry, CRH-positive BNST neurons that couple inflammatory and stress signals to sympathetic output, CeA/PVN CRH neurons linked to the splenic nerve, the classical HPA axis with ACTH as one endocrine arm, hypothalamic recall candidates including Otx2, Spp1/osteopontin, Slc18a2/VMAT2, Gpr88, and dopaminergic shifts, and a splenic decoding layer marked by Chrna3, Irf7, Isg15, Cxcl9, Cxcl10, Cxcl11, Ly6al, and the currently still uncharacterized upregulated transcript MOR5S4 (Hashimoto et al., 2026; Huerta et al., 2025; Jin et al., 2024; Kayyal et al., 2025; Koren et al., 2021; Rueckels and Picard-Maureau, 2025; Zhang et al., 2020). This model is broad enough to accommodate endocrine, sympathetic, and cholinergic relay mechanisms, yet specific enough to be falsified experimentally (Hashimoto et al., 2026; Hsueh et al., 1994; Huerta et al., 2025; Jin et al., 2024; Kayyal et al., 2025; Koren et al., 2021; Kuo et al., 2001; Rosas-Ballina et al., 2011; Rueckels and Picard-Maureau, 2025; Zhang et al., 2020).

## Several testable predictions follow

First, recall should show temporal propagation. Central activity should precede endocrine, autonomic, plasma mediator, and splenic transcriptional changes (Hashimoto et al., 2026; Kayyal et al., 2025; Koren et al., 2021; Rueckels and Picard-Maureau, 2025; Zhang et al., 2020). The approximately three-hour delay observed in the pilot transcriptomic study provides a working time window that can be tested with denser sampling (Rueckels and Picard-Maureau, 2025).

Second, efferent routes should be partially redundant but not interchangeable. Blocking ACTH or glucocorticoid-sensitive endocrine routing may impair NK-cell enhancement in one paradigm, whereas catecholamine depletion may be more important for neutrophil enhancement (Chao et al., 2005; Hsueh et al., 1994; Hsueh et al., 1999). CTL, antibody, NK, neutrophil, and macrophage-like effector readouts may therefore share central recall logic but diverge in peripheral implementation (Alvarez-Borda et al., 1995; Alvarez-Borda et al., 1999; Chao et al., 2005; Ghanta et al., 1987a; Ghanta et al., 1988; Ghanta et al., 1990; Hiramoto et al., 1993; Hsueh et al., 1994).

Third, hypothalamic recall candidates should be experimentally testable as upstream components of the efferent recall architecture. In particular, Otx2, Spp1/osteopontin, and Slc18a2/VMAT2 provide candidate entry points for testing whether transcriptional regulation, immune-neural matrix signaling, and monoaminergic vesicular transport contribute causally to conditioned immune recall rather than merely correlating with it (Rueckels and Picard-Maureau, 2025). Targeted manipulation of these candidates, combined with time-resolved endocrine, autonomic, plasma, and splenic readouts, should clarify whether the hypothalamus indeed functions as an upstream organizer of delayed peripheral recall.

Fourth, the spleen should contain cell-type-specific decoder populations. Single-cell and spatial transcriptomics should soon be able to identify whether Chrna3, Irf7, Cxcl9, Cxcl10, Cxcl11, Ly6al, MOR5S4, and related markers map to specific splenic immune-cell subsets, stromal compartments, vascular niches, or innervation-associated microenvironments (Rosas-Ballina et al., 2011; Rueckels and Picard-Maureau, 2025; Zhang et al., 2020).

Fifth, modern circuit tools should allow causal testing. Chemogenetic and/or optogenetic manipulation of insular, hypothalamic, amygdalar, BNST, PVN, RVLM, vagal, or splenic-nerve pathways should further be able to alter recall magnitude, timing, or immune specificity if these structures are part of the conditioned enhancement route (Ben-Shaanan et al., 2016; Ben-Shaanan et al., 2018; Hashimoto et al., 2026; Huerta et al., 2025; Jin et al., 2024; Kayyal et al., 2025; Koren et al., 2021; Zhang et al., 2020).

## Limitations of the current evidence

Several limitations are important to acknowledge. Much of the classical enhancement literature used small animal cohorts, older immune assays, and pharmacological tools with limited anatomical specificity (Chao et al., 2005; Ghanta et al., 1987a,b; Ghanta et al., 1988; Ghanta et al., 1990; Hiramoto et al., 1993; Hiramoto et al., 1987; Hsueh et al., 1994; Solvason et al., 1988; Solvason et al., 1989). Some tumor-model studies are difficult to compare directly with modern immuno-oncology standards, and many studies focused on male animals or did not systematically test sex as a biological variable (Ghanta et al., 1987a; Ghanta et al., 1988; Ghanta et al., 1990). Also, cue specificity, stress effects, odor salience, residual immune activation, handling effects, and nonspecific arousal are not always fully separable in older paradigms (Ghanta et al., 1987b; Hadamitzky et al., 2020; Rueckels and Picard-Maureau, 2025; Solvason et al., 1988).

Furthermore, gene-expression studies are mainly indicative and provide tissue-level transcriptional associations rather than cell-type-specific causal mechanisms (Rueckels and Picard-Maureau, 2025). Whether hypothalamic Otx2, Spp1, or Slc18a2, or splenic Chrna3, Irf7, Cxcl9, Cxcl10, Cxcl11, or even currently still uncharacterized transcripts as MOR5S4 are central players during recall remains to be proven, although providing a testable basis for future studies (Rueckels and Picard-Maureau, 2025).

Similarly, modern circuit studies also require careful interpretation. Insular immune-state retrieval, reward-system modulation of anti-tumor immunity, brain-to-spleen humoral control, cytokine-specific vagal coding, and BNST-PVN-RVLM inflammatory output are not the same experiment and accordingly should not be construed as one single pathway (Ben-Shaanan et al., 2016; Ben-Shaanan et al., 2018; Hashimoto et al., 2026; Huerta et al., 2025; Jin et al., 2024; Kayyal et al., 2025; Koren et al., 2021; Zhang et al., 2020). Their relevance is comparative and mechanistic: they show that the nervous system has multiple anatomically and molecularly defined routes for immune sensing, immune representation, and immune control (Ben-Shaanan et al., 2016; Ben-Shaanan et al., 2018; Hashimoto et al., 2026; Huerta et al., 2025; Jin et al., 2024; Kayyal et al., 2025; Koren et al., 2021; Zhang et al., 2020). The task for future conditioned enhancement studies is to determine which of these transcriptomic, endocrine, and neural routes are necessary, sufficient, or modulatory in specific immune-conditioning paradigms (Hsueh et al., 1994; Huerta et al., 2025; Jin et al., 2024; Kayyal et al., 2025; Koren et al., 2021; Kuo et al., 2001; Rueckels and Picard-Maureau, 2025; Zhang et al., 2020).

Finally, there is no established clinical protocol for conditioned immune enhancement in humans. Translational claims should therefore remain cautious (Hadamitzky et al., 2020; Schedlowski and Pacheco-López, 2010). At present, conditioned immune enhancement is best viewed as a biologically coherent and experimentally testable form of anticipatory neuroimmune recall, not as an established therapeutic intervention (Hadamitzky et al., 2020; Rueckels and Picard-Maureau, 2025; Schedlowski and Pacheco-López, 2010; Trabanelli et al., 2025; Table 1).

**Table 1:** Selected milestones in conditioned immune enhancement and efferent pathway mapping.

Study/year	Paradigm or technique	Acquisition or central representation	Efferent recall/immune readout	Main relevance
Metalnikov and Chorine (1926)	Pavlovian immune-reflex experiments in guinea pigs and rabbits	Immune-active stimulation paired with scratching or heat	Conditioned cue altered leukocyte profile; cue before infectious challenge produced enhancement-like resistance	Early anticipatory immune-conditioning concept
Ader and Cohen (1975)	Taste-aversion immune conditioning	Saccharin paired with cyclophosphamide	Cue re-exposure suppressed antibody response	Modern proof of learned immune modulation
Hiramoto et al. (1987)	Bidirectional NK conditioning	Different immune-active unconditioned stimuli paired with sensory cues	Cue recall enhanced or suppressed NK activity depending on the US	Directional programmability of immune recall
Ghanta et al. (1987a)	Tumor-bearing mice; conditioned natural immunity	Poly(I:C) paired with camphor odor	Increased survival in MOPC 104E myeloma-bearing mice	Preclinical tumor-model relevance
Ghanta et al. (1987b)	Camphor or saccharin-LiCl conditioning	Conditioned NK response tested against interferon conditioning	NK activity enhanced without uniformly conditioned interferon elevation	Early separation of NK enhancement from generic IFN elevation
Solvason et al. (1988)	Camphor odor or saccharin/LiCl paired with poly(I:C) or IFN-β	IFN-β, but not IFN-α, substituted for poly(I:C)	Cue recall enhanced NK activity	IFN-β as acquisition-related signal
Ghanta et al. (1988)	MOPC 104E tumor model	Viral-mimetic stimulation paired with odor	Delayed tumor growth and improved survival; macrophage-enriched splenic effects	Tumor-relevant conditioned enhancement
Solvason et al. (1989)	Opioid blockade during recall	Established camphor/poly(I:C) association	Naltrexone blocked conditioned NK elevation	Opioid-sensitive efferent gate
Hiramoto et al. (1990)	Reserpine intervention	Established conditioned NK paradigm	Reserpine disrupted retention or expression	Monoamine/catecholamine handling in recall
Ghanta et al. (1990)	YC8 lymphoma model	Camphor paired with allogeneic DBA/2 spleen-cell immunization	Camphor-only recall delayed tumor growth	Conditioned immunotherapeutic activity
Hiramoto et al. (1993)	CTL conditioning with camphor and allogeneic spleen cells	Allogeneic immunization paired with odor	Conditioned CTL recall blocked by centrally active naltrexone	Central opioid involvement in acquired cytotoxic immunity
Hsueh et al. (1994)	RIA for β-endorphin/ACTH; splenic IFN message	Camphor/poly(I:C) conditioning	ACTH and splenic IFN-α higher in conditioned animals	ACTH/IFN-α endocrine efferent model
Hsueh et al. (1995)	β-Endorphin intervention	Established conditioned NK paradigm	Conditioned NK expression was β-endorphin dependent	β-Endorphin as recall-stage mediator
Hsueh et al. (1996)	Opioid receptor subtype blockade	Established conditioned NK paradigm	μ-opioid receptor activation required	μ-opioid receptor requirement
Exton et al. (1998)	CsA-conditioned immunosuppression	Saccharin paired with cyclosporine A	Splenic innervation contributed to conditioned IL-2-related suppression	Anatomical efferent route in immunosuppression
Alvarez-Borda et al. (1999)	Antibody enhancement and lesion studies	Gustatory or odor CS paired with antigen	Insular cortex and amygdala lesions disrupted conditioned antibody enhancement	Central acquisition and representation structures
Hsueh et al. (1999)	Catecholamine blockade	Established conditioned NK paradigm	Dopaminergic and noradrenergic systems contributed to recall	Catecholaminergic recall mechanism
Kuo et al. (2001)	Neurotransmitter blockade	Previously acquired conditioned NK response	Glutamate/NMDA signaling required for recall	Excitatory recall gate
Hsueh et al. (2002)	Cholinergic and serotonergic manipulation	Established conditioned NK paradigm	Cholinergic and serotonergic systems required for conditioned NK response	Neurotransmitter-gated recall
Chao et al. (2005)	Conditioned neutrophil enhancement	Camphor/poly(I:C) conditioning	Reserpine and 6-OHDA blocked recall; dexamethasone did not	Catecholaminergic efference beyond NK cells
Ben-Shaanan et al. (2016)	Chemogenetic reward-system activation	Not classical conditioning	Enhanced innate and adaptive immunity	Central reward modulation of immune function
Ben-Shaanan et al. (2018)	Reward-system activation in tumor models	Not classical conditioning	Attenuated tumor growth through neuroimmune mechanisms	Central modulation of anti-tumor immunity
Zhang et al. (2020)	Optogenetic/chemogenetic brain-to-spleen mapping	Central modulation of humoral immunity	CeA/PVN CRH neurons, splenic nerve, ChAT+ T cells, and B-cell nicotinic signaling regulated antibody responses	Causal splenic efferent pathway outside HPA-only model
Mahmud et al. (2020)	Osteopontin/SPP1 in chronic viral neuroinflammation	Not conditioning	OPN/SPP1 behaved as a molecular brake on neuroinflammation	Biological context for hypothalamic Spp1/OPN recall signal
Koren et al. (2021)	FosTRAP/chemogenetic immune-state retrieval	Peripheral inflammation encoded in insular cortex	Reactivation retrieved peripheral immune states	Central immune-state representation
Jin et al. (2024)	Body-brain inflammatory circuit mapping	Cytokine-specific vagal afferent coding	Brainstem circuits shaped inflammatory balance	Afferent cytokine coding relevant to acquisition
Kayyal et al. (2025)	Conditioned immune retrieval circuit	Conditioned taste-immune association	Bidirectional anterior-posterior insula circuit supported conditioned immune retrieval	Circuit-level conditioned immune retrieval
Rueckels and Picard-Maureau (2025)	Multi-tissue transcriptomic and plasma pilot study	Camphor/poly(I:C) conditioning	Hypothalamic Otx2-Spp1-Slc18a2/VMAT2 and splenic Chrna3-Irf7-Cxcl9/10/11 signatures; delayed recall pattern	Candidate efferent recall map
Huerta et al. (2025)	In vivo cytokine imaging in vagal sensory neurons	Cytokine-specific nodose ganglion coding	Distinct real-time vagal representations of cytokine signals	Afferent immune-state encoding relevant to future conditioning models
Trabanelli et al. (2025)	Virtual infection-threat paradigm in humans	Anticipatory perception of infectious avatars	Salience-network engagement and innate lymphoid-cell changes	Human anticipatory neuroimmune response outside classical conditioning
Hashimoto et al. (2026)	IL-1β-responsive central neurons	Peripheral IL-1β encoded centrally	BNST-PVN-RVLM β-adrenergic inflammatory output	Parallel sympathetic efferent inflammatory pathway

## Conclusion

The efferent pathway hypothesis provides a framework for understanding conditioned immune enhancement as a learned neuroimmune recall process rather than as a simple reactivation of the original immune stimulus (Hadamitzky et al., 2020; Hsueh et al., 1994; Rueckels and Picard-Maureau, 2025; Solvason et al., 1988; Solvason et al., 1989). The historical trajectory is important in this respect. Early immune-conditioning experiments already included enhancement-like resistance to infection, while later work mostly established conditioned immunosuppression as the dominant translational branch (Ader and Cohen, 1975; Goebel et al., 2002; Hadamitzky et al., 2020; Metalnikov and Chorine, 1926; Schedlowski and Pacheco-López, 2010). Research by Hiramoto, Ghanta, Solvason, Hsueh, and colleagues then showed that immune enhancement can be experimentally conditioned, recalled, pharmacologically blocked, and extended from NK activity to CTL responses, neutrophil activity, antibody responses, and tumor-model readouts (Alvarez-Borda et al., 1995; Alvarez-Borda et al., 1999; Chao et al., 2005; Ghanta et al., 1987a,b; Ghanta et al., 1988; Ghanta et al., 1990; Hiramoto et al., 1993; Hiramoto et al., 1987; Hsueh et al., 1994; Solvason et al., 1988; Solvason et al., 1989).

The central insight emerging from this literature is that acquisition, central storage, and efferent recall are in deed separable biological processes (Hadamitzky et al., 2020; Hsueh et al., 1994; Huerta et al., 2025; Jin et al., 2024; Kayyal et al., 2025; Koren et al., 2021; Rueckels and Picard-Maureau, 2025; Solvason et al., 1988). As the efferent pathways are the focus of this review, available evidence argues against a model in which conditioned immune enhancement is mediated simply by reactivation of the hypothalamic–pituitary–adrenal axis (Hsueh et al., 1994; Rueckels and Picard-Maureau, 2025). The HPA axis remains important, particularly through ACTH, CRH-related signaling, and splenic regulation; however, the classical enhancement literature already identified additional recall-stage components, including endogenous opioids, *β*-endorphin-related peptides, *μ*-opioid receptors, glutamate/NMDA signaling, monoamine and catecholamine handling, and cholinergic and serotonergic activity (Hiramoto et al., 1990; Hsueh et al., 1994; Hsueh et al., 1995; Hsueh et al., 1996; Hsueh et al., 1999; Hsueh et al., 2002; Kuo et al., 2001; Solvason et al., 1989). Thus, even before modern circuit tools became available, the efferent pathway was already emerging as a multi-channel architecture rather than a single endocrine reflex (Hsueh et al., 1994; Hsueh et al., 1999; Kuo et al., 2001; Solvason et al., 1989).

Recent circuit-level studies now make this interpretation more plausible and more experimentally tractable. Immune states can be represented and later retrieved through the insula, conditioned retrieval can depend on an anterior–posterior insula circuit, humoral responses can be regulated through CeA/PVN CRH-related brain-to-spleen pathways, and inflammatory physiology can be routed centrally through CRH-positive BNST neurons and a BNST→PVN → RVLM→*β*-adrenergic pathway (Hashimoto et al., 2026; Kayyal et al., 2025; Koren et al., 2021; Zhang et al., 2020). At the same time, afferent cytokine information is now known to be encoded by distinct vagal sensory populations in the nodose ganglia, with gene-expression data adding an additional hypothesis-generating molecular layer (Huerta et al., 2025; Jin et al., 2024; Rueckels and Picard-Maureau, 2025).

Conditioned immune enhancement therefore currently remains less explored yet increasingly converges with findings from conditioned immunosuppression, both representing biologically coherent forms of anticipatory neuroimmune recall (Hadamitzky et al., 2020; Kayyal et al., 2025; Koren et al., 2021; Rueckels and Picard-Maureau, 2025; Schedlowski and Pacheco-López, 2010; Trabanelli et al., 2025). The next experimental step is to test this multi-route efferent architecture directly: define the afferent signals that establish immune learning, identify the central representations that store the cue-immune association, and map the efferent routes that execute recall in specific tissues and immune-cell compartments (Hsueh et al., 1994; Huerta et al., 2025; Jin et al., 2024; Kayyal et al., 2025; Koren et al., 2021; Kuo et al., 2001; Rueckels and Picard-Maureau, 2025; Zhang et al., 2020). Future studies should combine receptor blockade, endocrine interruption, nerve manipulation, optogenetic and chemogenetic circuit tools, plasma mediator profiling, cytokine imaging, single-cell or spatial transcriptomics, and functional immune assays (Ben-Shaanan et al., 2016; Ben-Shaanan et al., 2018; Hashimoto et al., 2026; Huerta et al., 2025; Jin et al., 2024; Kayyal et al., 2025; Koren et al., 2021; Kuo et al., 2001; Rueckels and Picard-Maureau, 2025; Zhang et al., 2020).

Deciphering how the mind autonomously orchestrates systemic restoration through its multiple efferent pathways remains one if not the ‘holy grail’ of psychosomatic medicine. It marks the critical step from a mere descriptive observation of anecdotal evidence to a precise intervention: harnessing the power of the mind to heal the body by leveraging the central nervous system and its messengers as a precision instrument to actively modulate systemic immunity as an effective complement for existing therapies.
