# Correction: The efferent pathway hypothesis—A mini-review on conditioned immune enhancement

**DOI:** 10.3389/fnhum.2026.1963726

**Published:** 2026-08-21

**Authors:** Markus Rueckels, Srinivas Reddy Boreddy, Marcus Picard-Maureau

**Affiliations:** Lisa Kolk Foundation, Leverkusen, Germany

**Keywords:** anticipatory immunity, CAIP, Chrna3, conditioned immune enhancement, efferent pathway, osteopontin, Otx2, VMAT2

Madden, K. S., Boehm, G. W., Lee, S. C., Grota, L. J., Cohen, N., and Ader, R. (2001). One-trial conditioning of the antibody response to hen egg lysozyme in rats. *J. Neuroimmunol*. 113, 236–239. doi: 10.1016/S0165-5728(00)00448-3

and

Madden, K. S., Boehm, G. W., Lee, S. C., Grota, L. J., Cohen, N., and Ader, R. (2000). One-trial conditioning of the primary antibody response to hen egg lysozyme in rats. *Brain Behav. Immunity* 14, 113–114.

were not cited in the article.

The citations have now been inserted in the Section **Extension beyond NK cells**, paragraph 3, and should read:

“Conditioned enhancement of antibody responses provides a further extension. Additional one-trial conditioning work by Madden and colleagues showed conditioned enhancement of the antibody response to hen egg lysozyme in rats, further supporting antibody production as an immune-effector domain in which conditioned enhancement can be observed (Madden et al., 2000; Madden et al., 2001). Antigen-paired taste or odor cues can enhance later antibody production, and lesion studies indicate that insular cortex and amygdala lesions disrupt conditioned antibody enhancement, whereas hippocampal lesions do not show the same effect (Alvarez-Borda et al., 1995; Alvarez-Borda et al., 1999). These data pointed toward central acquisition and representation structures long before modern engram and circuit tools became available (Alvarez-Borda et al., 1995; Alvarez-Borda et al., 1999; Kayyal et al., 2025; Koren et al., 2021).”

The original version of this article has been updated.

