# Supplementary figures and images for: The efferent pathway hypothesis—A mini-review on conditioned immune enhancement
Source: Front Hum Neurosci. 2026 Jun 25;20:1845376. doi: 10.3389/fnhum.2026.1845376 (PMC13346077; doi:10.3389/fnhum.2026.1845376)

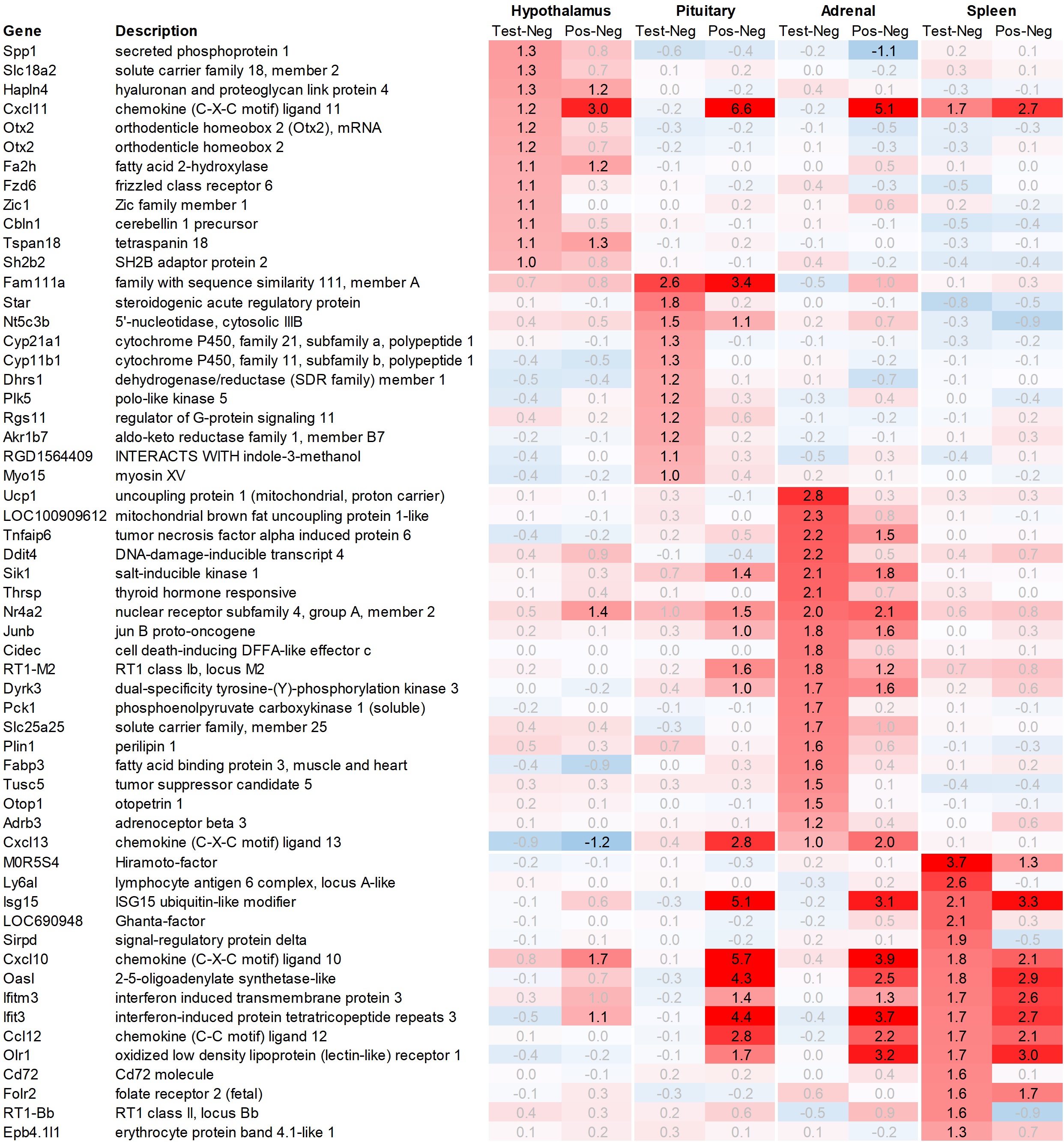

Supplement: Supplementary file 1 [file Image_1.JPEG]

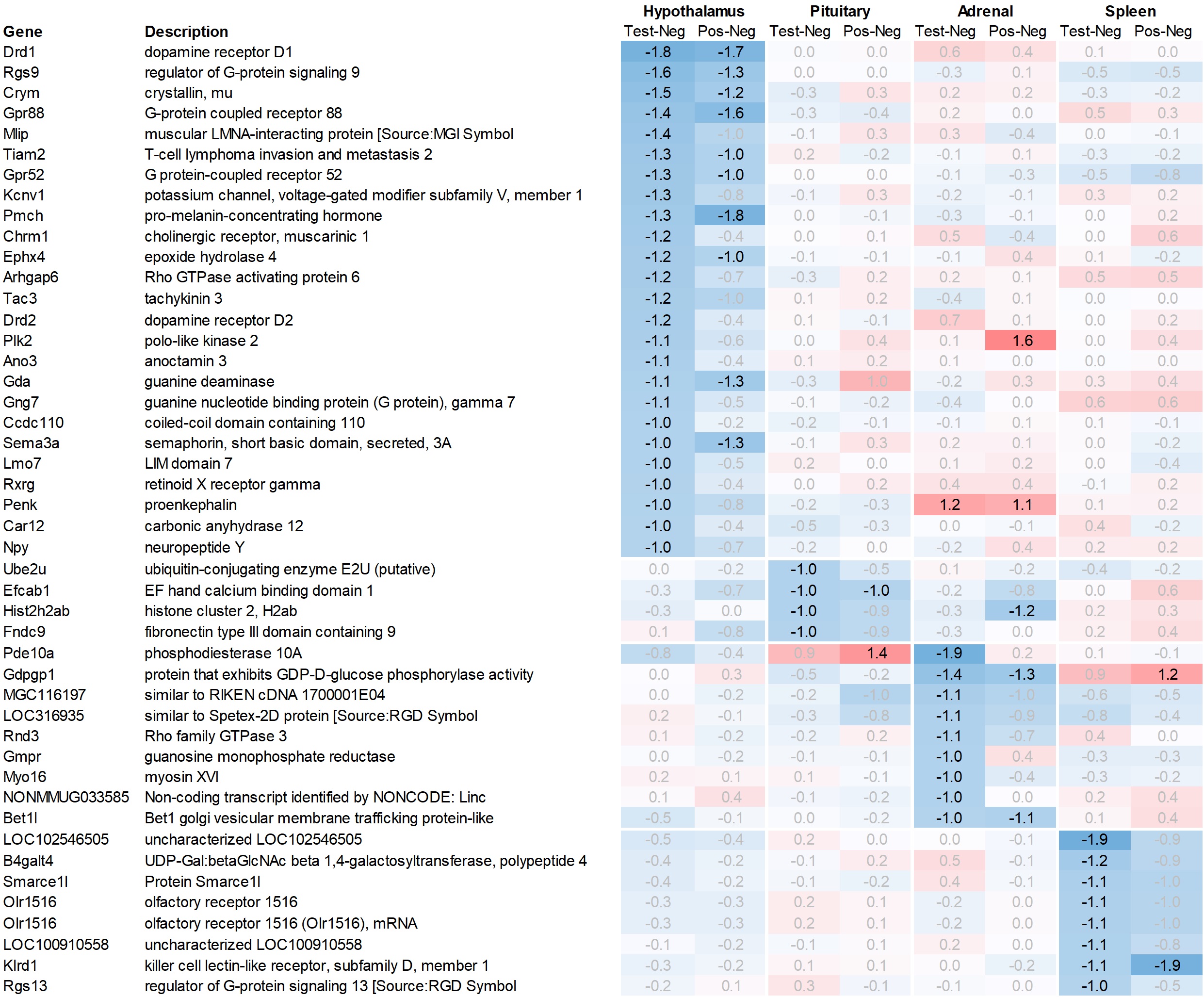

Supplement: Supplementary file 2 [file Image_2.JPEG]
